# Supplementary material for: Assessing the spatial structure of the association between attendance at preschool and children’s developmental vulnerabilities in Queensland, Australia
Source: PLoS One. 2023 Aug 9;18(8):e0285409. doi: 10.1371/journal.pone.0285409 (PMC10411799; doi:10.1371/journal.pone.0285409)
Supplement: S1 Appendix — (PDF) [file pone.0285409.s001.pdf]

S1 Appendix. Moran's I and local R^2.

A Moran's I

Moran's I [2] can be calculated as

$$I = \frac{n \sum_{i=1}^{n} \sum_{j=1}^{n} w_{i,j} z_i z_j}{S \sum_{i=1}^{n} z_i^2}, \quad (1)$$

where $z_i = x_i - \overline{x}$ and $S = \sum_{i=1}^{n} \sum_{j=1}^{n} w_{i,j}$. Here, x_i is the independent variable, $\overline{x}$ is the associated sample mean and w_{ij} is an element of the spatial matrix S, which shows the degree of spatial connection between regions i and j [1].

B Local R^2

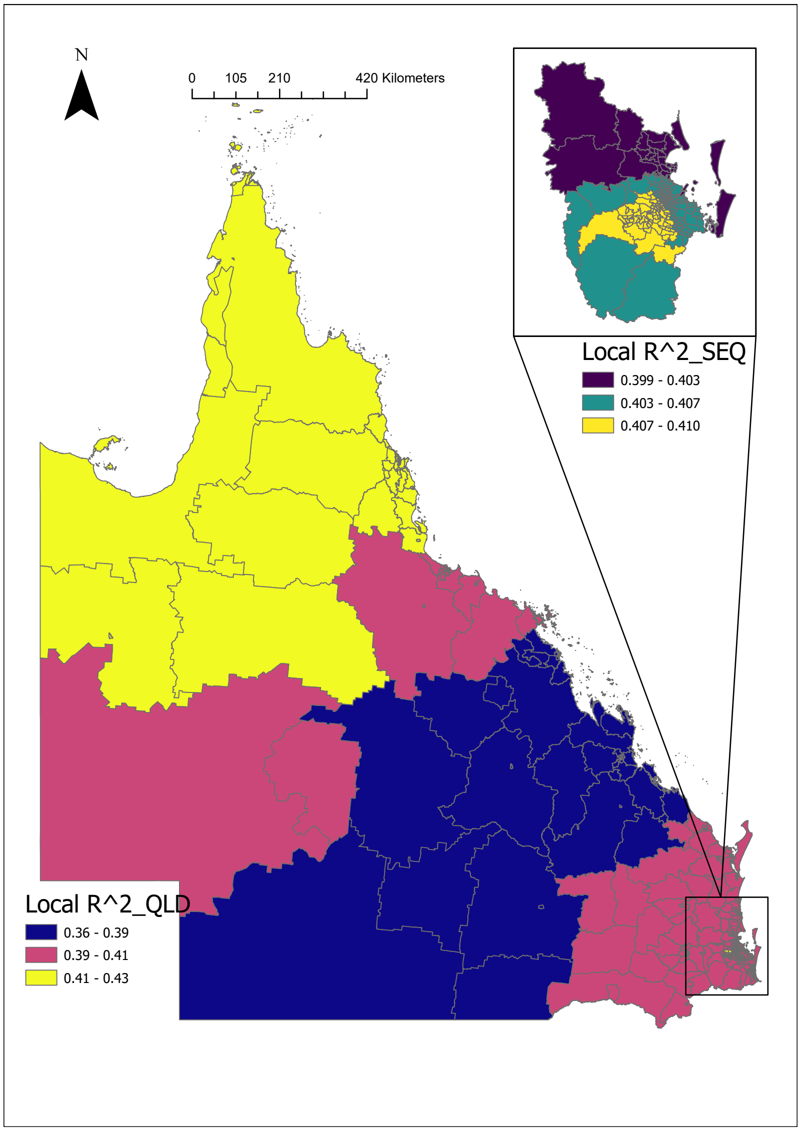

Figure B1: The spatial distribution of local R^2 values of the GWR model.

References

[1] S. Kalogirou and T. Hatzichristos. A spatial modelling framework for income estimation. *Spatial Economic Analysis*, 2(3):297–316, 2007.

[2] P. Moran. The interpretation of statistical maps. *Journal of the Royal Statistical Society: Series B (Methodological)*, 10(2):243–251, 1948.
