# Supplementary material for: Assessing the spatial structure of the association between attendance at preschool and children’s developmental vulnerabilities in Queensland, Australia
Source: PLoS One. 2023 Aug 9;18(8):e0285409. doi: 10.1371/journal.pone.0285409 (PMC10411799; doi:10.1371/journal.pone.0285409)
Supplement: S4 Appendix — (PDF) [file pone.0285409.s004.pdf]

S4 Appendix. Relationship between Indigenous and other socio-demographic variables.

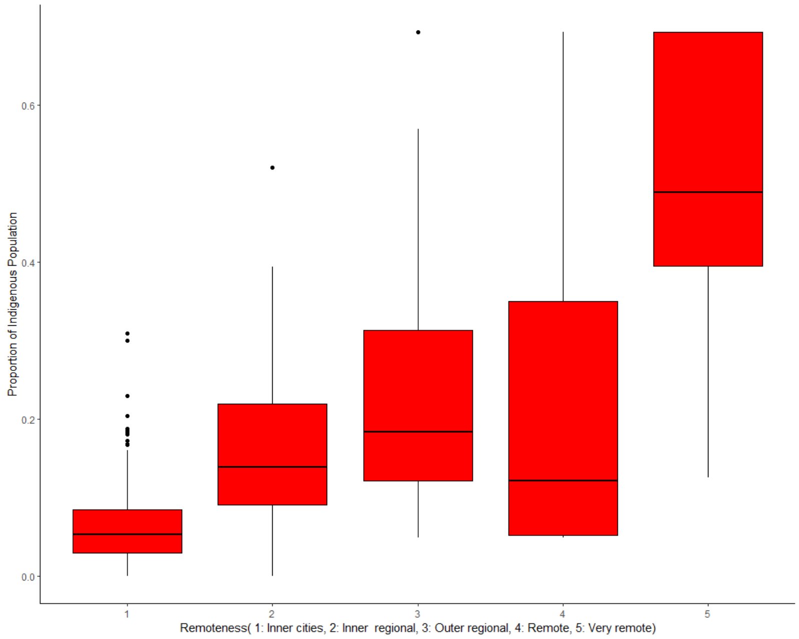

Figure 1: A box plot showing the relationship between the levels of remoteness (Inner cities, Inner regional, Outer regional, Remote, Very remote) and the proportion of people with Indigenous status at SA2 level.

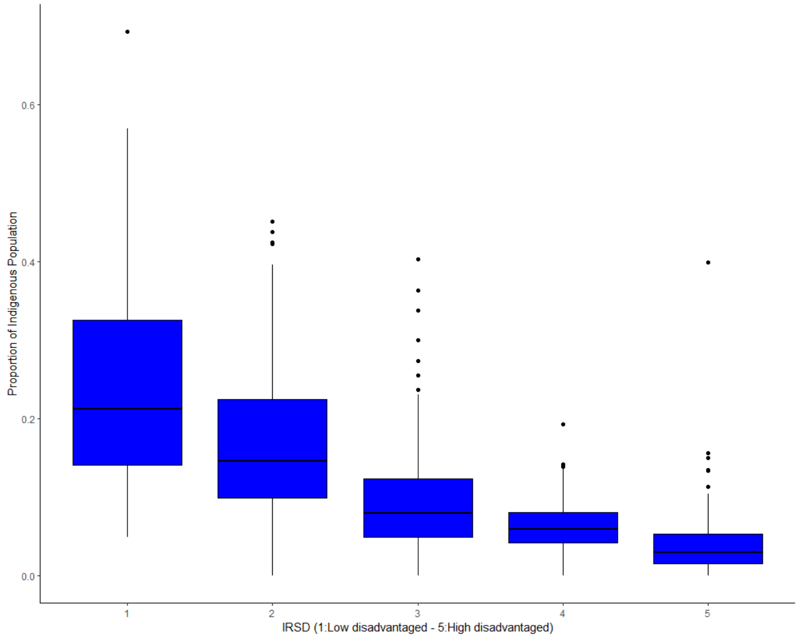

Figure 2: A box plot showing the distribution of the IRSD factor and the proportion of Indigenous population status at the SA2 level. A higher proportion of the Indigenous population is in the most disadvantaged SA2 regions.
