# Supplementary material for: Assessing the spatial structure of the association between attendance at preschool and children’s developmental vulnerabilities in Queensland, Australia
Source: PLoS One. 2023 Aug 9;18(8):e0285409. doi: 10.1371/journal.pone.0285409 (PMC10411799; doi:10.1371/journal.pone.0285409)
Supplement: S6 Appendix — (PDF) [file pone.0285409.s006.pdf]

S1 Appendix. Additional analysis

A Moran's I

Moran's I [2] can be calculated as

$$I = \frac{n \sum_{i=1}^{n} \sum_{j=1}^{n} w_{i,j} z_i z_j}{S \sum_{i=1}^{n} z_i^2}, \tag{1}$$

where $z_i = x_i - \overline{x}$ and $S = \sum_{i=1}^{n} \sum_{j=1}^{n} w_{i,j}$. Here, x_i is the independent variable, $\overline{x}$ is the associated sample mean and w_{ij} is an element of the spatial matrix S, which shows the degree of spatial connection between regions i and j [1].

B Local R^2

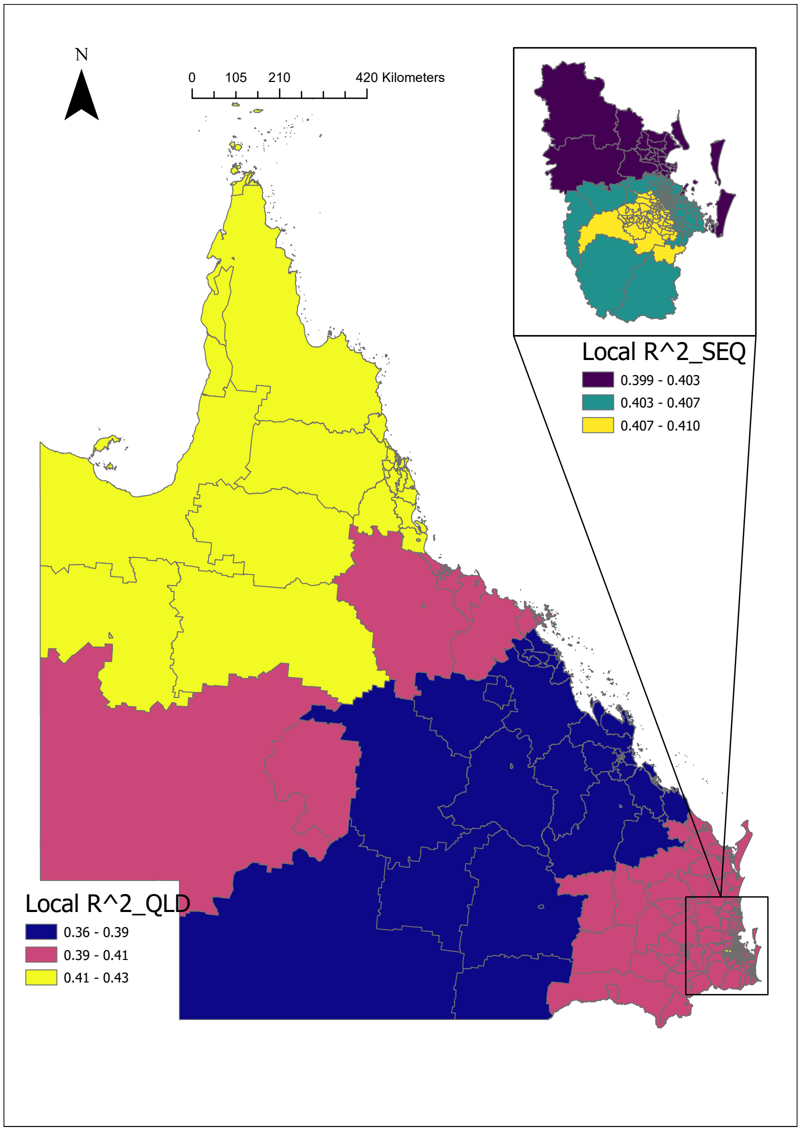

Figure B1: The spatial distribution of local R^2 values of the GWR model

C Clusters inside Greater Brisbane

As depicted in Figure 2 from the main paper, there was a substantial spatial variation among the SA2s in the Greater Brisbane region. The global Moran's I for Vuln 1 was 0.351, indicating an overall significant positive spatial autocorrelation between Greater Brisbane SA2 areas. Table C1 shows summary statistics of the GWR coefficients for the 236 SA2 in greater Brisbane which summarize the mean, and the range of GWR coefficients and shows the global P-value from OLS model. Appendix D shows the local t-value for SA2 area in Greater Brisbane. From this table, the most significant variables are the IRSD, attendance at preschool and remoteness (inner regional). The K-means cluster analysis identified two clusters (Figure C1). Cluster 1 includes around 150 SA2 areas which covered almost of the Grater Brisbane areas; cluster 2 includes around 86 areas. Figure C2 shows the box plot for GWR coefficients inside each cluster.

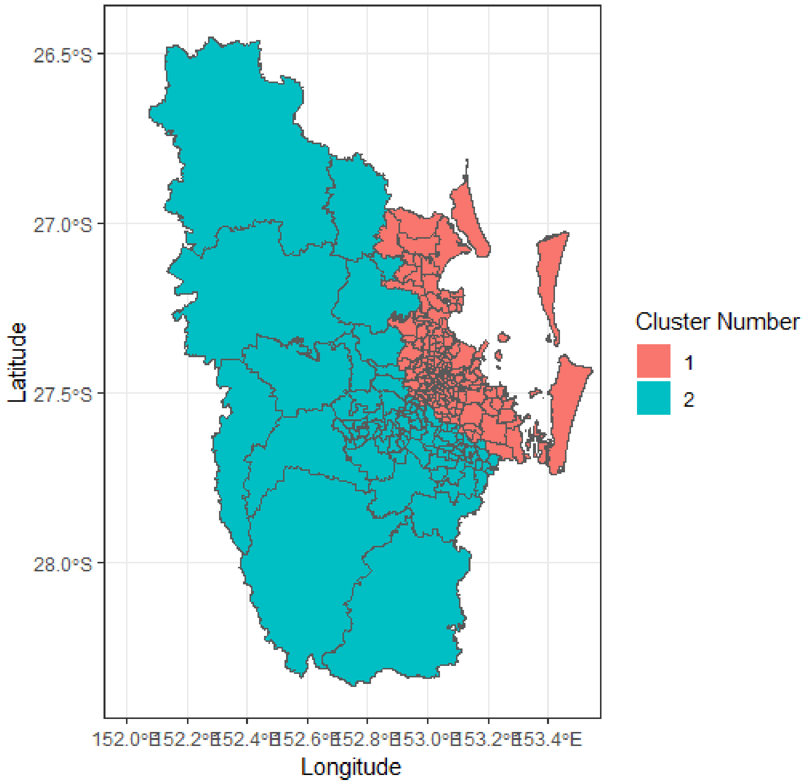

Figure C1: Clustering of GWR coefficients in spatial distribution map in Greater Brisbane

Cluster 1 is the most differentiated from the other cluster by generally larger negative GWR coefficients for the outer regional factor, cluster 2 has a small negative relationship with Vuln 1 in term of remote factor. Clusters 1 and 2 demonstrate that when the socioeconomic factor increases (move from the most disadvantages level 1 to the least disadvantages areas level 5) the proportion of Vuln 1 decreases. Moreover, when the remoteness increases (move from major cities to the remote areas), the proportion of Vuln 1 also increases.

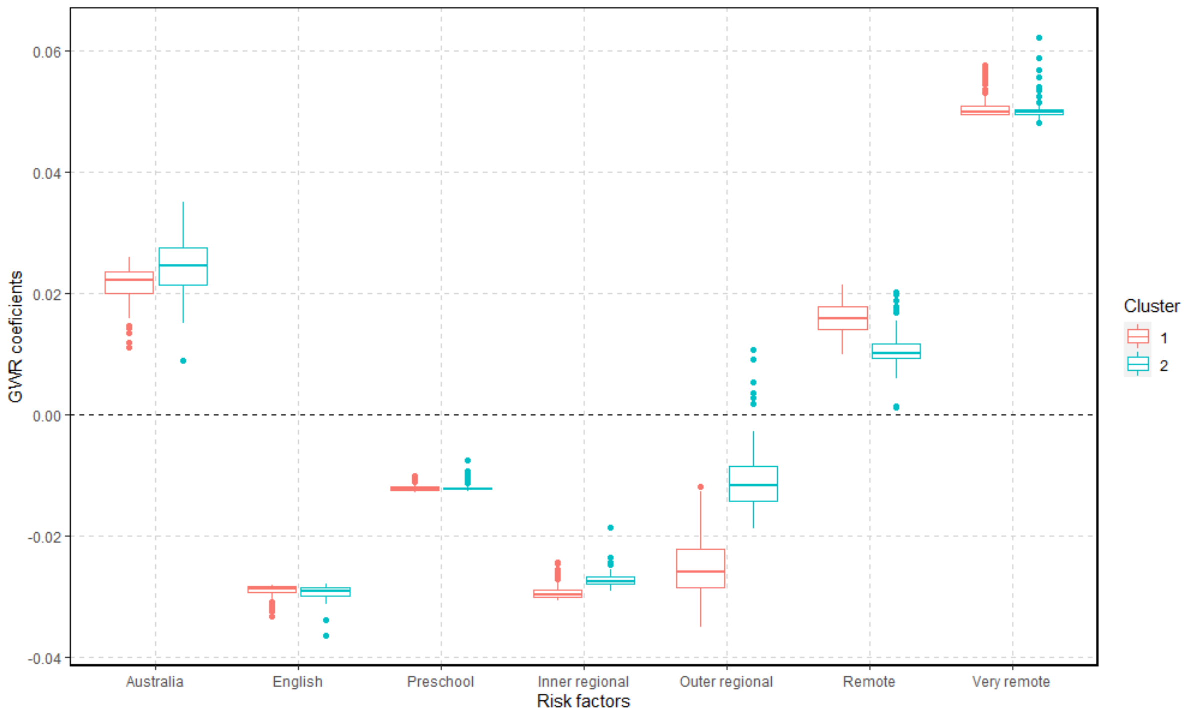

Figure C2: A comparison of the GWR coefficients of Vuln 1 and risk factors in each Queensland cluster within Greater Brisbane

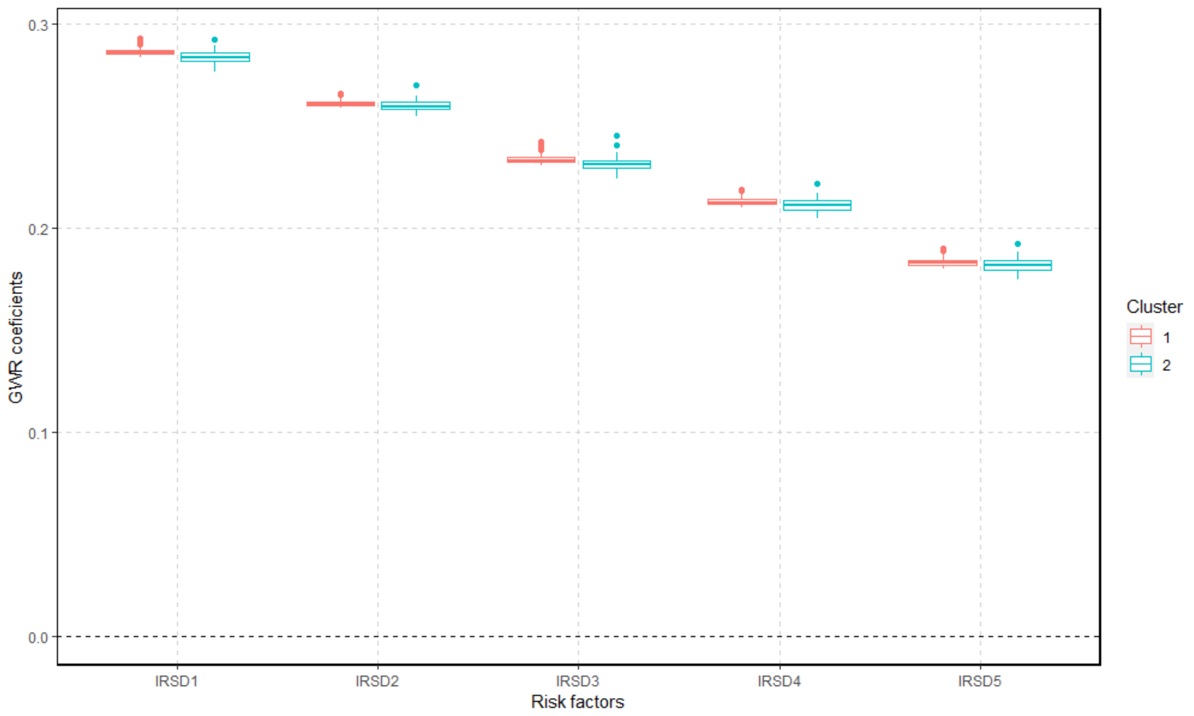

Figure C3: A comparison of the GWR coefficients of Vuln 1 and risk factors in each Queensland cluster within Greater Brisbane

Table C1: GWR model coefficients for greater Brisbane, with P-value from OLS model.

Explanatory variables	Mean	Range	P-value
Preschool	-0.012	[-0.013, -0.008]	0.016
English	-0.029	[-0.037, -0.028]	0.504
Australia	0.023	[0.009, 0.035]	0.614
IRSD (Quintile 1)	0.285	[0.276, 0.293]	9.11e-13
IRSD (Quintile 2)	0.285	[0.276, 0.293]	2.09e-10
IRSD (Quintile 3)	0.26	[0.254, 0.270]	1.51e-08
IRSD (Quintile 4)	0.212	[0.204, 0.221]	3.20e-07
IRSD (Quintile 5)	0.182	[0.175, 0.192]	8.77e-06
Remoteness (Inner regional)	-0.029	[-0.031, -0.018]	0.001
Remoteness (Outer regional)	-0.020	[-0.035, 0.011]	0.304
Remoteness (Remote)	0.014	[0.001, 0.021]	0.691
Remoteness (Very remote)	0.050	[0.047, 0.062]	0.991

D Summary of GWR coefficients

Table D1: Number of significant regions in Queensland from GWR coefficients significant is based on $|t| < 2$.

Explanatory variables	$t < -2$	$\|t\| < 2$	$t > 2$
Attendance at preschool	386	139	0
Australia is the country of birth	1	525	0
English as a mother language	126	399	0
IRSD (Quintile 1)	0	1	525
IRSD (Quintile 2)	0	1	525
IRSD (Quintile 3)	0	1	525
IRSD (Quintile 4)	0	1	525
IRSD (Quintile 5)	0	1	525
Remoteness (Inner regional)	319	206	0
Remoteness (Outer regional)	1	525	0
Remoteness (Remote)	1	525	0
Remoteness (Very remote)	1	421	104

Table D2: Number of significant regions in Greater Brisbane from GWR coefficients significant is based on $|t| < 2$.

Explanatory variables	$t < -2$	$\|t\| < 2$	$t > 2$
Attendance at preschool	235	1	0
English as a mother language	1	235	0
English	1	235	0
IRSD (Quintile 1)	0	1	235
IRSD (Quintile 2)	0	1	235
IRSD (Quintile 3)	0	1	235
IRSD (Quintile 4)	0	1	235
IRSD (Quintile 5)	0	1	235
Remoteness (Inner regional)	236	0	0
Remoteness (Outer regional)	1	235	0
Remoteness (Remote)	1	235	0
Remoteness (Very remote)	1	235	0

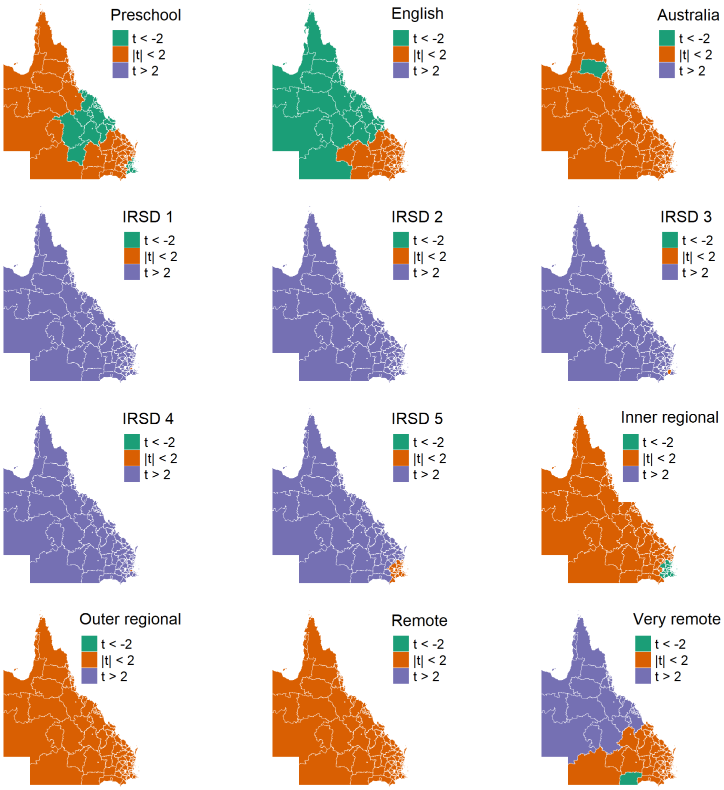

Figure D1: The geographical distribution of significant and non significant coefficients from the GWR analysis of the SA2 regions in Queensland based on $|t| < 2$.

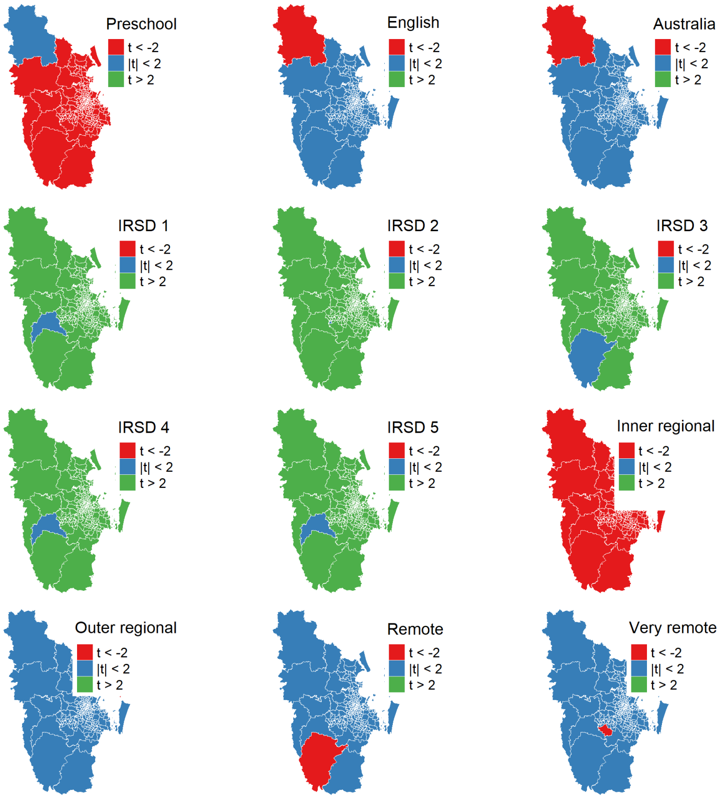

Figure D2: The spatial distribution of significant coefficients from GWR with Greater Brisbane.

E Silhouette score

Number of clusters (K)	Silhouette score
**3**	**0.72**
4	0.52
5	0.51
6	0.53
7	0.51
8	0.54
9	0.54
10	0.54

Table E1: Silhouette scores of different K values for Vuln 1 in QLD.

Number of clusters (K)	Silhouette score
2	0.43
3	0.44
4	0.44
5	0.34
6	0.35
7	0.37
8	0.34
9	0.34

Table E2: Silhouette scores of different K values for Vuln 1 in Greater Brisbane.

F Relation between Indigenous and other socio-demographic variables, IRSD and remoteness.

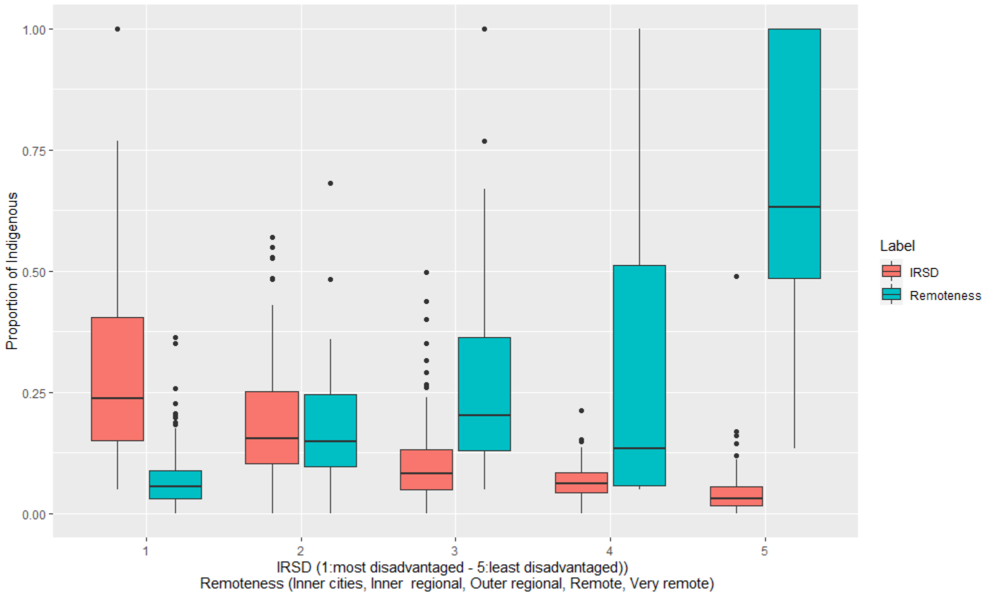

Figure F1: Box-plot between remoteness factor, socioeconomic factor and the proportion of Indigenous status.

G GWR coefficients for each type of AEDC domain

Table G1: Cluster size for each type of AEDC domain and the average coefficients for attendance at preschool in the first cluster.

Domain	C1	C2	C3	Preschool
Physical	374	89	63	-0.10
Social	286	144	96	-0.20
Emotional	369	106	51	-0.09
Language	387	105	34	-0.08
Communication	276	156	94	-0.14

Table G2: GWR average coefficients from the adaptive and fixed kernel for each type of AEDC domains

Domain	Physical		Social		Emotional		Language		Communication		Vuln 1		Vuln 2	
Parameter	adaptive	fixed	adaptive	fixed	adaptive	fixed	adaptive	fixed	adaptive	fixed	adaptive	fixed	adaptive	fixed
Quasi global R^2	0.29	0.27	0.29	0.28	0.18	0.17	0.54	0.53	0.42	0.40	0.42	0.39	0.42	0.40
AICs	-1778.8	-1773.8	-1778.8	-1854.6	-1781.8	-1980.6	-2084.8	-2080.4	-1943.8	-1918.6	-1600.1			-1793.0
average GWR coefficients														
Preschool	-0.007	-0.007	-0.007	-0.008	-0.004	-0.004	-0.007	-0.005	-0.008	-0.007	-0.010	-0.007	-0.012	-0.008
English	0.011	0.012	0.004	0.007	0.004	0.005	-0.12	-0.005	-0.092	-0.095	-0.041	-0.50	-0.002	0.014
Austrila	0.007	0.006	-0.31	-0.03	-0.015	-0.015	0.012	-0.019	0.013	0.005	0.002	-0.022	-0.013	-0.017
IRSD (Quintile 1)	0.137	0.137	0.16	0.158	0.127	0.126	0.108	0.007	0.174	0.179	0.301	0.318	0.183	0.151
IRSD (Quintile 2)	0.113	0.114	0.136	0.134	0.109	0.109	0.088	0.112	0.164	0.167	0.275	0.291	0.157	0.189
IRSD (Quintile 3)	0.096	0.096	0.127	0.125	0.103	0.102	0.067	0.092	0.141	0.146	0.249	0.267	0.137	0.164
IRSD (Quintlie 4)	0.089	0.087	0.118	0.116	0.098	0.097	0.057	0.071	0.131	0.136	0.23	0.250	0.121	0.143
IRSD (Quintile 5)	0.007	0.077	0.101	0.099	0.089	0.089	0.042	0.063	0.116	0.122	0.202	0.224	0.105	0.129
Remoteness (Inner regional)	0.002	0.002	-0.014	-0.014	-0.007	-0.007	-0.005	0.002	-0.02	-0.009	-0.019	-0.008	-0.021	-0.009
Remoteness (Outer regional)	-0.001	-0.001	-0.008	-0.008	-0.011	-0.012	-0.002	0.007	-0.043	-0.01	-0.012	-0.007	-0.29	-0.008
Remoteness (Remote)	-0.001	0.006	-0.013	-0.015	-0.019	-0.022	0.023	0.029	0.02	0.012	0.008	0.006	-0.009	-0.004
Remoteness (Very remote)	0.005	0.032	-0.013	-0.022	-0.002	-0.008	0.054	0.055	0.10	0.043	0.049	0.03	0.03	0.017

References

[1] S. Kalogirou and T. Hatzichristos. A spatial modelling framework for income estimation. *Spatial Economic Analysis*, 2(3):297–316, 2007.

[2] P. Moran. The interpretation of statistical maps. *Journal of the Royal Statistical Society: Series B (Methodological)*, 10(2):243–251, 1948.
