# Supplementary material for: Assessing the spatial structure of the association between attendance at preschool and children’s developmental vulnerabilities in Queensland, Australia
Source: PLoS One. 2023 Aug 9;18(8):e0285409. doi: 10.1371/journal.pone.0285409 (PMC10411799; doi:10.1371/journal.pone.0285409)
Supplement: S1 Data — (ZIP) [file pone.0285409.s007.zip › Appendix/S2_final.pdf]

S2 Appendix. Clusters inside Greater Brisbane and Summary of GWR coefficients

Clusters inside Greater Brisbane

As depicted in Fig 2 from the main paper, there was substantial spatial variation among the SA2s in the Greater Brisbane region. The global Moran's I for Vuln 1 was 0.351, indicating an overall significant positive spatial autocorrelation between Greater Brisbane SA2 areas.

Table 1 shows the mean, range, and P-value for the coefficients of the GWR model for Greater Brisbane. The coefficients represent the relationship between the independent variables (Preschool, English, Australia, IRSD quintiles 1-5, and remoteness levels including Inner Regional, Outer Regional, Remote, and Very Remote) and Vuln 1. The range column displays the range of the coefficients across the regions within Greater Brisbane. In contrast, the P-value column shows the significance of the relationship between the independent and dependent variables. A lower P-value indicates a stronger relationship and a higher level of significance. According to the table, the IRSD quintiles 1-5 and Inner Regional remoteness level have a significant relationship with Vuln 1, while the other independent variables have a weak relationship with the Vuln 1.

A K-means cluster analysis was performed to identify the spatial distribution of GWR coefficients in Greater Brisbane. The analysis resulted in two clusters (Fig 1), with cluster 1 covering almost all of the Greater Brisbane areas and including around 150 SA2 regions and cluster 2 including around 86 regions. The box plots for GWR coefficients inside each cluster can be seen in Fig 2 and Fig 3. These visualizations provide a way to compare the distribution of the coefficients within each cluster and highlight any significant differences between the two clusters.

Fig 1. A visualization of the spatial distribution of GWR coefficients in Greater Brisbane, grouped into clusters using a K-means analysis.

Cluster 1 is the most differentiated from cluster 2 by generally larger negative GWR coefficients for the outer regional factor. Cluster 2 has a small negative relationship with Vuln 1 in term of the remoteness factor. Clusters 1 and 2 demonstrate that when the socioeconomic factor increases (moves from most disadvantaged areas (level 1) to the least disadvantaged areas (level 5) the proportion of Vuln 1 decreases. Moreover, when remoteness increases (moves from major cities to remote areas), the proportion of Vuln 1 also increases.

Tables 2 and 3 provide an overview of the number of SA2 regions in Queensland and Greater Brisbane with significant GWR coefficients at a significance level of 0.05. The tables display the count of regions where the t-value falls below -2, between -2 and 2, and above 2. A t-value greater than 2 or less than -2 assume statistical significance. The spatial distribution of these regions is depicted in Fig 4 and Fig5 for Queensland and Greater Brisbane, respectively.

Fig 2. A comparison of the coefficients for Vuln 1 and the risk factors (Australia, English, Preschool, and remoteness including inner regional, outer regional, remote, and very remote levels) obtained from the GWR model in the two clusters within Greater Brisbane.

Fig 3. A comparison of the GWR coefficients for the relationship between Vuln 1 and its associated risk factor, the Index of Relative Socio-economic Disadvantage (IRSD), across the five levels in the two clusters within Greater Brisbane. The IRSD has five levels, with level 1 being the most disadvantaged.

Table 1. GWR model coefficients for Greater Brisbane, with P-value from OLS model.

Explanatory variables	Mean	Range	P-value
Preschool	-0.012	[-0.013, -0.008]	0.016
English	-0.029	[-0.037, -0.028]	0.504
Australia	0.023	[0.009, 0.035]	0.614
IRSD (Quintile 1)	0.285	[0.276, 0.293]	9.11e-13
IRSD (Quintile 2)	0.285	[0.276, 0.293]	2.09e-10
IRSD (Quintile 3)	0.26	[0.254, 0.270]	1.51e-08
IRSD (Quintile 4)	0.212	[0.204, 0.221]	3.20e-07
IRSD (Quintile 5)	0.182	[0.175, 0.192]	8.77e-06
Remoteness (Inner regional)	-0.029	[-0.031, -0.018]	0.001
Remoteness (Outer regional)	-0.020	[-0.035, 0.011]	0.304
Remoteness (Remote)	0.014	[0.001, 0.021]	0.691
Remoteness (Very remote)	0.050	[0.047, 0.062]	0.991

Summary of GWR coefficients

Table 2. The table provides the number of significant SA2 regions in Queensland with GWR coefficients at a significant level of 0.05, where $|t| > 2$ indicates statistical significance.

Explanatory variables	$t < -2$	$\|t\| < 2$	$t > 2$
Attendance at preschool	386	139	0
Australia is the country of birth	1	525	0
English as a mother language	126	399	0
IRSD (Quintile 1)	0	1	525
IRSD (Quintile 2)	0	1	525
IRSD (Quintile 3)	0	1	525
IRSD (Quintile 4)	0	1	525
IRSD (Quintile 5)	0	1	525
Remoteness (Inner regional)	319	206	0
Remoteness (Outer regional)	1	525	0
Remoteness (Remote)	1	525	0
Remoteness (Very remote)	1	421	104

Fig 4. The spatial distribution of significant coefficients from a GWR model with SA2 regions in Queensland which visualization the relationship between the Vuln 1 and the independent variables in different regions within Queensland. The significance of the coefficients is determined by the t-value, where regions with $|t| > 2$ are considered significant.

Fig 5. The spatial distribution of significant coefficients from a GWR model with SA2 regions in Greater Brisbane which show the relationship between Vuln 1 and the independent variables in different regions within Greater Brisbane. The t-value determines the significance of the coefficients, and regions with $|t| > 2$ are considered to be significantly different from zero at a given level of significance of 0.05.

Table 3. The table provides the number of significant SA2 regions in Greater Brisbane with GWR coefficients at a significant level of 0.05, where $|t| > 2$ indicates statistical significance.

Explanatory variables	$t < -2$	$\|t\| < 2$	$t > 2$
Attendance at preschool	235	1	0
English as a mother language	1	235	0
English	1	235	0
IRSD (Quintile 1)	0	1	235
IRSD (Quintile 2)	0	1	235
IRSD (Quintile 3)	0	1	235
IRSD (Quintile 4)	0	1	235
IRSD (Quintile 5)	0	1	235
Remoteness (Inner regional)	236	0	0
Remoteness (Outer regional)	1	235	0
Remoteness (Remote)	1	235	0
Remoteness (Very remote)	1	235	0
