# Supplementary material for: Assessing the spatial structure of the association between attendance at preschool and children’s developmental vulnerabilities in Queensland, Australia
Source: PLoS One. 2023 Aug 9;18(8):e0285409. doi: 10.1371/journal.pone.0285409 (PMC10411799; doi:10.1371/journal.pone.0285409)
Supplement: S1 Data — (ZIP) [file pone.0285409.s007.zip › Appendix/S3_final_.pdf]

S3 Appendix. Additional analysis.

A Silhouette score

Number of clusters (K)	Silhouette score
2	0.70
**3**	**0.72**
4	0.61
5	0.57
6	0.58
7	0.54
8	0.55
9	0.53
10	0.52

Table A1. Silhouette scores for different numbers of clusters (K) for Vuln 1 in Queensland. The table indicates the Silhouette score for each value of K, with the highest score of 0.72 being achieved for K = 3.

Number of clusters (K)	Silhouette score
2	0.44
3	0.44
4	0.44
5	0.34
6	0.35
7	0.37
8	0.34
9	0.34

Table A2. Silhouette scores for various numbers of clusters (K) for Vuln 1 in Greater Brisbane. The table lists the Silhouette score for each value of K, with the highest score of 0.44 being achieved for $K = 2$, 3, and 4.

B GWR coefficients for each type of AEDC domain

Table B1. Cluster size for each type of AEDC domain for the 3 clusters (C1, C2, C3), and the average coefficients for attendance at preschool in the first cluster for each type of health domain.

Domain	C1	C2	C3	Preschool
Physical	374	89	63	-0.10
Social	286	144	96	-0.20
Emotional	369	106	51	-0.09
Language	387	105	34	-0.08
Communication	276	156	94	-0.14

Table B2. GWR average coefficients from the adaptive and fixed kernel for each type of AEDC domains.

Domain	Physical		Social		Emotional		Language		Communication		Vuln 1		Vuln 2	
Kernel	adaptive	fixed	adaptive	fixed	adaptive	fixed	adaptive	fixed	adaptive	fixed	adaptive	fixed	adaptive	fixed
Quasi global R^2	0.29	0.27	0.29	0.28	0.18	0.17	0.54	0.53	0.42	0.40	0.42	0.39	0.42	0.40
AIC	-1778.8	-1773.8	-1778.8	-1854.6	-1781.8	-1980.6	-2084.8	-2080.4	-1943.8	-1918.6	-1600.1	-1670.6	-1788.8	-1793.0
average GWR coefficients														
Preschool	-0.007	-0.007	-0.007	-0.008	-0.004	-0.004	-0.007	-0.005	-0.008	-0.007	-0.010	-0.007	-0.012	-0.008
English	0.011	0.012	0.004	0.007	0.004	0.005	-0.12	-0.005	-0.092	-0.095	-0.041	-0.50	-0.002	0.014
Australia	0.007	0.006	-0.31	-0.03	-0.015	-0.015	0.012	-0.019	0.013	0.005	0.002	-0.022	-0.013	-0.017
IRSD (Quintile 1)	0.137	0.137	0.16	0.158	0.127	0.126	0.108	0.007	0.174	0.179	0.301	0.318	0.183	0.151
IRSD (Quintile 2)	0.113	0.114	0.136	0.134	0.109	0.109	0.088	0.112	0.164	0.167	0.275	0.291	0.157	0.189
IRSD (Quintile 3)	0.096	0.096	0.127	0.125	0.103	0.102	0.067	0.092	0.141	0.146	0.249	0.267	0.137	0.164
IRSD (Quintlie 4)	0.089	0.087	0.118	0.116	0.098	0.097	0.057	0.071	0.131	0.136	0.23	0.250	0.121	0.143
IRSD (Quintile 5)	0.007	0.077	0.101	0.099	0.089	0.089	0.042	0.063	0.116	0.122	0.202	0.224	0.105	0.129
Remoteness (Inner regional)	0.002	0.002	-0.014	-0.014	-0.007	-0.007	-0.005	0.002	-0.02	-0.009	-0.019	-0.008	-0.021	-0.009
Remoteness (Outer regional)	-0.001	-0.001	-0.008	-0.008	-0.011	-0.012	-0.002	0.007	-0.043	-0.01	-0.012	-0.007	-0.29	-0.008
Remoteness (Remote)	-0.001	0.006	-0.013	-0.015	-0.019	-0.022	0.023	0.029	0.02	0.012	0.008	0.006	-0.009	-0.004
Remoteness (Very remote)	0.005	0.032	-0.013	-0.022	-0.002	-0.008	0.054	0.055	0.10	0.043	0.049	0.03	0.03	0.017
