# Supplementary material for: Assessing the spatial structure of the association between attendance at preschool and children’s developmental vulnerabilities in Queensland, Australia
Source: PLoS One. 2023 Aug 9;18(8):e0285409. doi: 10.1371/journal.pone.0285409 (PMC10411799; doi:10.1371/journal.pone.0285409)
Supplement: S1 Data — (ZIP) [file pone.0285409.s007.zip › Appendix/S3_PVOD.pdf]

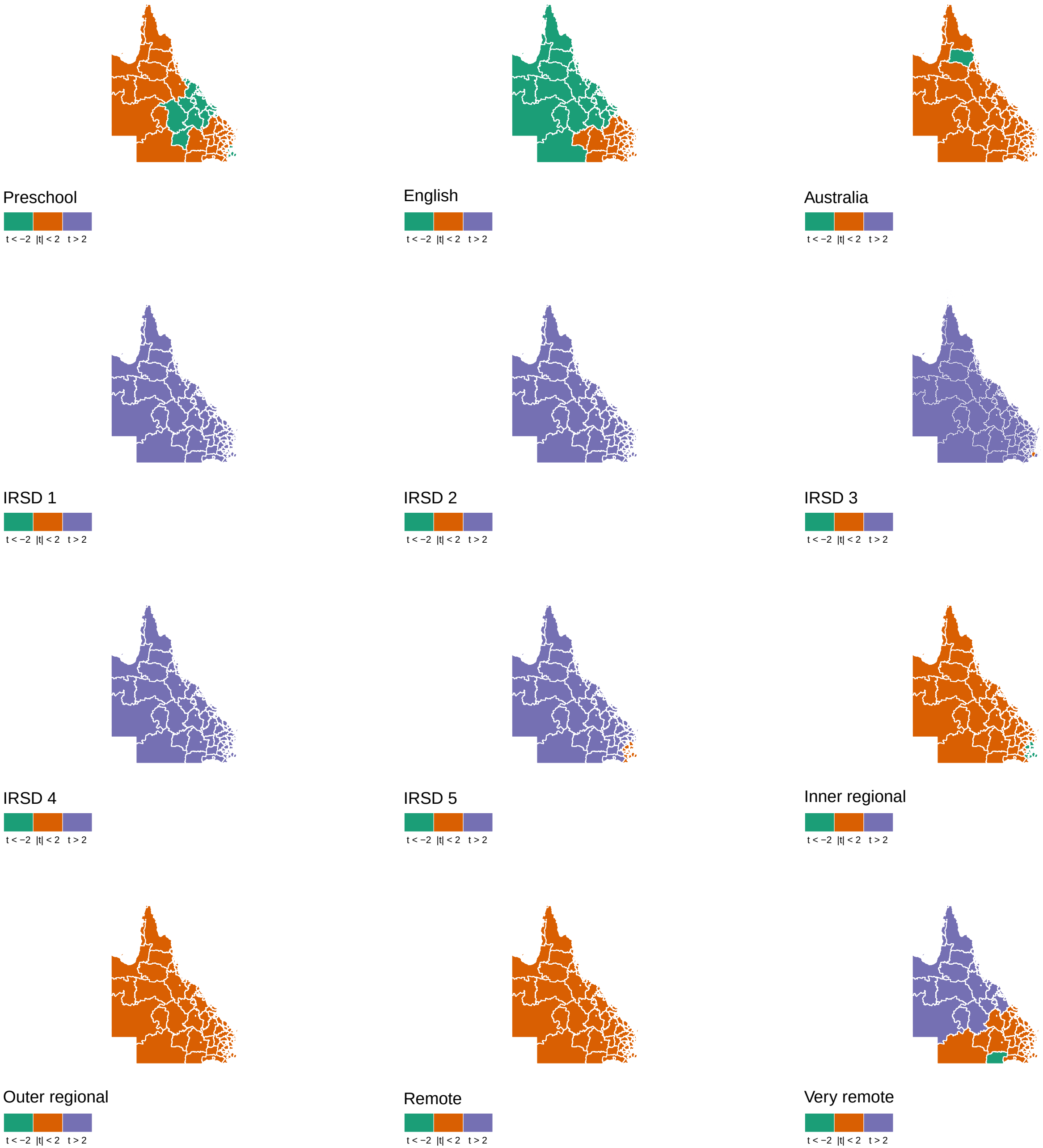
Preschool
t < −2 |t| < 2 t > 2
English
t < −2 |t| < 2 t > 2
Australia
t < −2 |t| < 2 t > 2
IRSD 1
t < −2 |t| < 2 t > 2
IRSD 2
t < −2 |t| < 2 t > 2
IRSD 3
t < −2 |t| < 2 t > 2
IRSD 4
t < −2 |t| < 2 t > 2
IRSD 5
t < −2 |t| < 2 t > 2
Inner regional
t < −2 |t| < 2 t > 2
Outer regional
t < −2 |t| < 2 t > 2
Remote
t < −2 |t| < 2 t > 2
Very remote
t < −2 |t| < 2 t > 2
