# Supplementary material for: Assessing the spatial structure of the association between attendance at preschool and children’s developmental vulnerabilities in Queensland, Australia
Source: PLoS One. 2023 Aug 9;18(8):e0285409. doi: 10.1371/journal.pone.0285409 (PMC10411799; doi:10.1371/journal.pone.0285409)
Supplement: S1 Data — (ZIP) [file pone.0285409.s007.zip › Appendix/S5_final.pdf]

S5 Appendix. Cluster Accuracy

Three different clustering algorithms were selected to investigate and compare the clustering of GWR coefficients. The algorithms that were chosen are the K-means algorithm, hierarchical clustering, and partition around medoids (PAM). Comparisons are based on four general criteria: popularity, flexibility, applicability and ability to handle high-dimensional data [1].

The algorithms are summarised below:

K-means algorithm

K-meas is the most common unsupervised algorithm. The algorithm proceeds as follows [2]: 1) Define the number of clusters, K. 2) Randomly select K data points as the cluster centroids. 3) Assign data points to the closest cluster centroid. 4) Recompute the cluster centroids. 5) Repeat steps 3) and 4) until either the centroids do not change or the maximum number of iterations is reached.

Hierarchical clustering algorithm

The hierarchical clustering algorithm finds the clusters in an agglomerative or divisive manner. In this algorithm, each data point begins in its own cluster and then combines the like pair of clusters gradually, resulting in a hierarchical structure. The hierarchical clustering algorithm is conducted as follows [7]: 1) Each observation is assumed as an initial cluster. 2) Calculate the distances between groups. 3) Two clusters with the shortest distance between them are combined and replaced with a single cluster. The distance matrix is then recomputed to account for the merging operation. 4) Repeat Steps 2 and 3 until only one cluster contains all observations.

Partition around medoids (PAM)

The key distinction between the K-means and PAM method is that K-means employs centroids, but PAM employs medoids, which are always the real points in the dataset. This algorithm proceeds as follows [9]: 1) Choose K random observations for an initial set of medoids. 2) Assign each data point to the nearest medoid using Euclidean distance or another method. 3) Start to increase clustering quality by swapping selected observations with unselected observations. 4) Repeat steps 2 and 3 until the average distance between items and medoids is the minimum.

Validation and comparison

Accuracy is usually used to measure the quality of classification, which is also used for clustering. Several measures have been developed to validate the findings of a clustering study and determine which clustering method performs best for a certain experiment. This validation might be based only on the data's internal properties or on some external reference [5, 6, 11]. In this paper, different methods were adopted to validate the clusters using internal validation, including connectivity [3], average silhouette width [10], and Dunn index [8]. The connectivity was determined using K-nearest neighbours, which measures the degree of connectedness of the clusters. Connectivity indicates how closely related the items in the same cluster are in the data space. The

silhouette width ranges from -1 (poorly clustered observations) to 1 (well-clustered observations) and measures the compactness and separation of the clusters. The Dunn index is a measure of the ratio between the smallest distance between observations in different clusters and the largest intra-cluster distance. The value of the Dunn index ranges from 0 to infinity, with higher values indicating better clustering results.

The validation was run using the clvalid package in R [4]. A summary of results from the three algorithms is found in Table 1. In this table, the search for the optimal number of clusters was between 2 to 10. This table shows that the optimal number of clusters for the GWR coefficients is 3. The geographic distributions of these clusters are also depicted on the map of Queensland in Fig 1.

Table 1. The comparison between different clustering algorithms was performed based on the measures of connectivity, silhouette width, and Dunn index.

method		2	3	4	5	6	7	8	9	10
	Connectivity	10.2	9.17	17.9	23.4	30.6	58.5	59.2	75.7	77.3
K-means	Dunn	0.080	0.051	0.077	0.081	0.046	0.027	0.027	0.021	0.018
	Silhouette	0.709	0.717	0.611	0.579	0.581	0.547	0.554	0.528	0.517
	connectivity	6.9	7.7	14.4	15.7	18.2	26.8	28.8	33.4	36.3
Hierarchical	Dunn	0.081	0.114	0.065	0.065	0.051	0.051	0.051	0.051	0.051
	Silhouette	0.709	0.719	0.606	0.582	0.580	0.555	0.553	0.539	0.484
	Connectivity	12.4	9.2	40.23	44.2	53.3	71.4	77.3	84.3	91.5
PAM	Dunn	0.027	0.051	0.013	0.019	0.019	0.017	0.017	0.018	0.004
	Silhouette	0.708	0.717	0.553	0.562	0.566	0.531	0.532	0.505	0.490

Fig 1. The geographical distribution of three clusters using K-means, hierarchical, and partition around medoids (PAM) algorithms.

Table 2. Cluster sizes (number of SA2 regions in each cluster) for 3 clusters for each of the clustering algorithms considered.

	C1	C2	C3
K-means algorithm	342	104	80
Hierarchical algorithm	352	104	70
PAM algorithm	342	104	80

Table 2 provides the number of SA2 regions in each cluster for the different methods. Cluster 1 covers more than half of the regions in Queensland, including Greater Brisbane.

The comparison between the algorithms is made through comparative confusion matrices and accuracy rates. The confusion matrix shows the different combinations of predicted and actual values and is used to calculate precision and accuracy. Since the actual cluster labels are not available, the confusion matrix is calculated by using the results from the cluster algorithms as either actual or predicted values, depending on the comparison. The results can be found in Table 3.

Table 3. The confusion matrix between (K-means-PAM), (K-means-Hierarchical), and (PAM-Hierarchical), where the accuracy rate between (K-means-PAM) is 1, The accuracy rate between(K-means-Hierarchical) is 0.98, and the accuracy rate between (PAM-Hierarchical) is 0.98.

		PAM (Actual)		
		C1	C2	C3
	C1	342	0	0
K-means (Predicted)	C2	0	104	0
	C3	0	0	80

		Hierarchical (Actual)		
		C1	C2	C3
	C1	342	0	0
K-means (Predicted)	C2	0	104	0
	C3	10	0	70

		Hierarchical (Actual)		
		C1	C2	C3
	C1	342	0	0
PAM (Predicted)	C2	0	104	0
	C3	10	0	70

These results indicate that the clustering algorithms are highly consistent with each other, with all three having accuracy rates above 0.98. The high accuracy rates suggest that there is a high agreement between the results from each of the clustering algorithms and that the subgroups identified are robust across different methodologies. This supports the argument that the clustering algorithms effectively uncover meaningful subgroups within the data.

References

1. O. A. Abbas. Comparisons between data clustering algorithms. *International Arab Journal of Information Technology (IAJIT)*, 5(3), 2008.

2. M. Ahmed, R. Seraj, and S. M. S. Islam. The k-means algorithm: A comprehensive survey and performance evaluation. *Electronics*, 9(8):1295, 2020.

3. M. R. Brito, E. L. Chávez, A. J. Quiroz, and J. E. Yukich. Connectivity of the mutual k-nearest-neighbor graph in clustering and outlier detection. *Statistics & Probability Letters*, 35(1):33–42, 1997.

4. G. Brock, V. Pihur, S. Datta, and S. Datta. clvalid: An R package for cluster validation. *Journal of Statistical Software*, 25:1–22, 2008.

5. S. Datta and S. Datta. Methods for evaluating clustering algorithms for gene expression data using a reference set of functional classes. *BMC bioinformatics*, 7(1):1–9, 2006.

6. F. D. Gibbons and F. P. Roth. Judging the quality of gene expression-based clustering methods using gene annotation. *Genome research*, 12(10):1574–1581, 2002.

7. F. Murtagh and P. Contreras. Algorithms for hierarchical clustering: an overview. *Wiley Interdisciplinary Reviews: Data Mining and Knowledge Discovery*, 2(1):86–97, 2012.

8. C.-E. B. Ncir, A. Hamza, and W. Bouaguel. Parallel and scalable dunn index for the validation of big data clusters. *Parallel Computing*, 102:102751, 2021.

9. H.-S. Park and C.-H. Jun. A simple and fast algorithm for k-medoids clustering. *Expert systems with applications*, 36(2):3336–3341, 2009.

10. K. R. Shahapure and C. Nicholas. Cluster quality analysis using silhouette score. In *2020 IEEE 7th International Conference on Data Science and Advanced Analytics (DSAA)*, pages 747–748. IEEE, 2020.

11. K. Y. Yeung, D. R. Haynor, and W. L. Ruzzo. Validating clustering for gene expression data. *Bioinformatics*, 17(4):309–318, 2001.
