# Supplementary figures and images for: Assessing the spatial structure of the association between attendance at preschool and children’s developmental vulnerabilities in Queensland, Australia
Source: PLoS One. 2023 Aug 9;18(8):e0285409. doi: 10.1371/journal.pone.0285409 (PMC10411799; doi:10.1371/journal.pone.0285409)

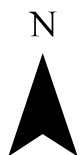

0 105 210 420 Kilometers

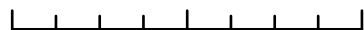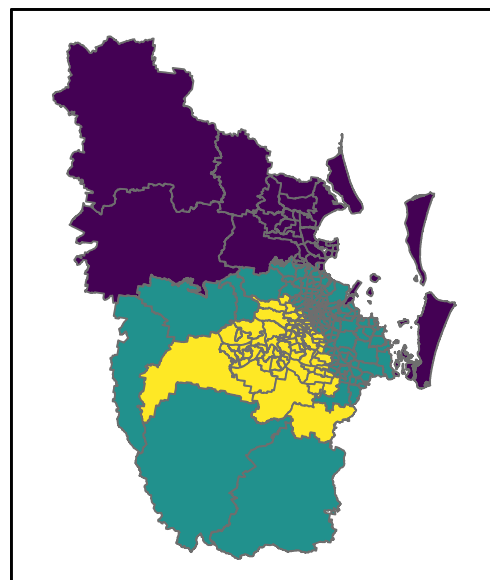

Local  $R^2_{SEQ}$

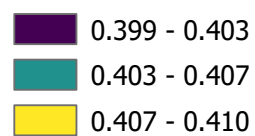

Local  $R^2_{QLD}$

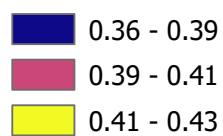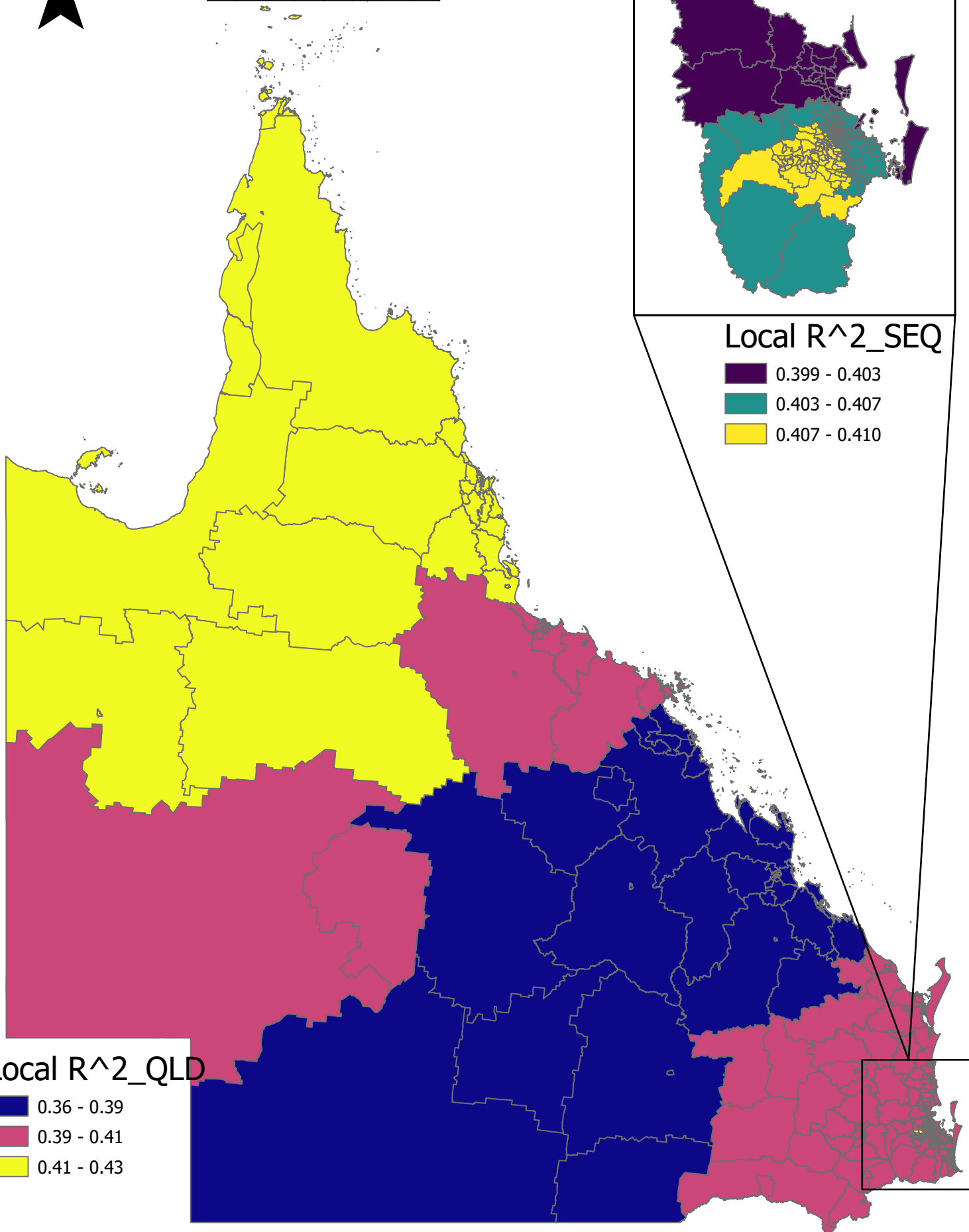

Supplement: S1 Data — (ZIP) [file pone.0285409.s007.zip › Appendix/S1_fig_B1.pdf]

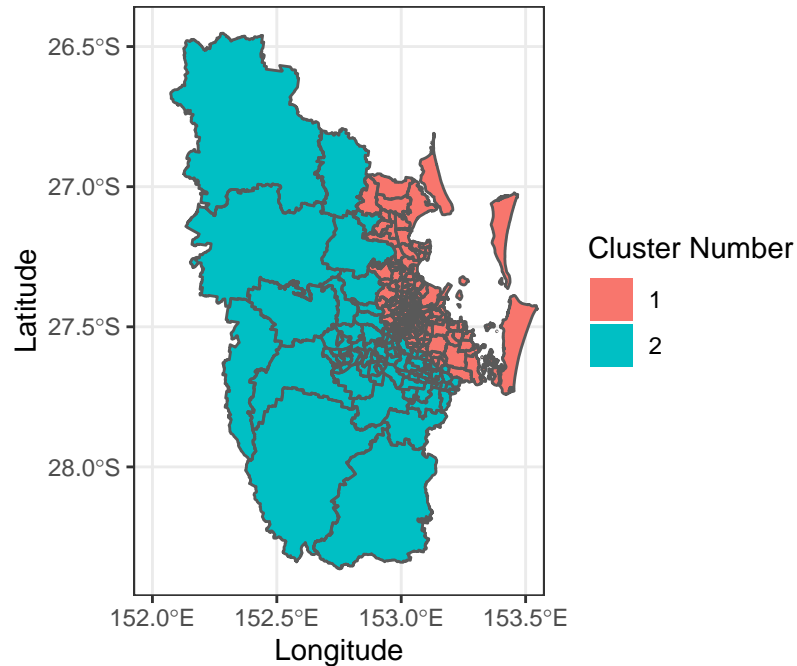

Supplement: S1 Data — (ZIP) [file pone.0285409.s007.zip › Appendix/S2_fig_1.pdf]

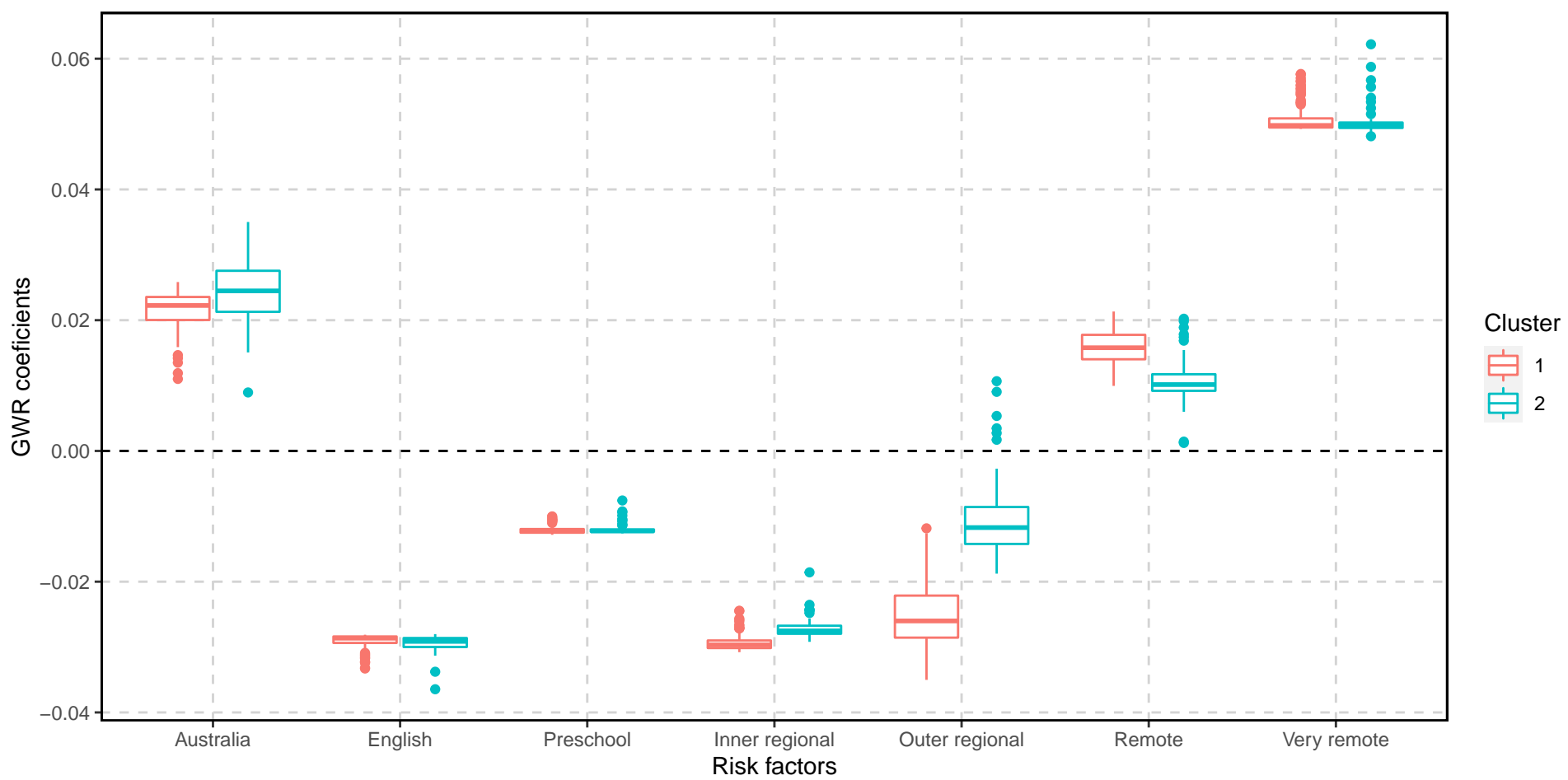

Supplement: S1 Data — (ZIP) [file pone.0285409.s007.zip › Appendix/S2_fig_2.pdf]

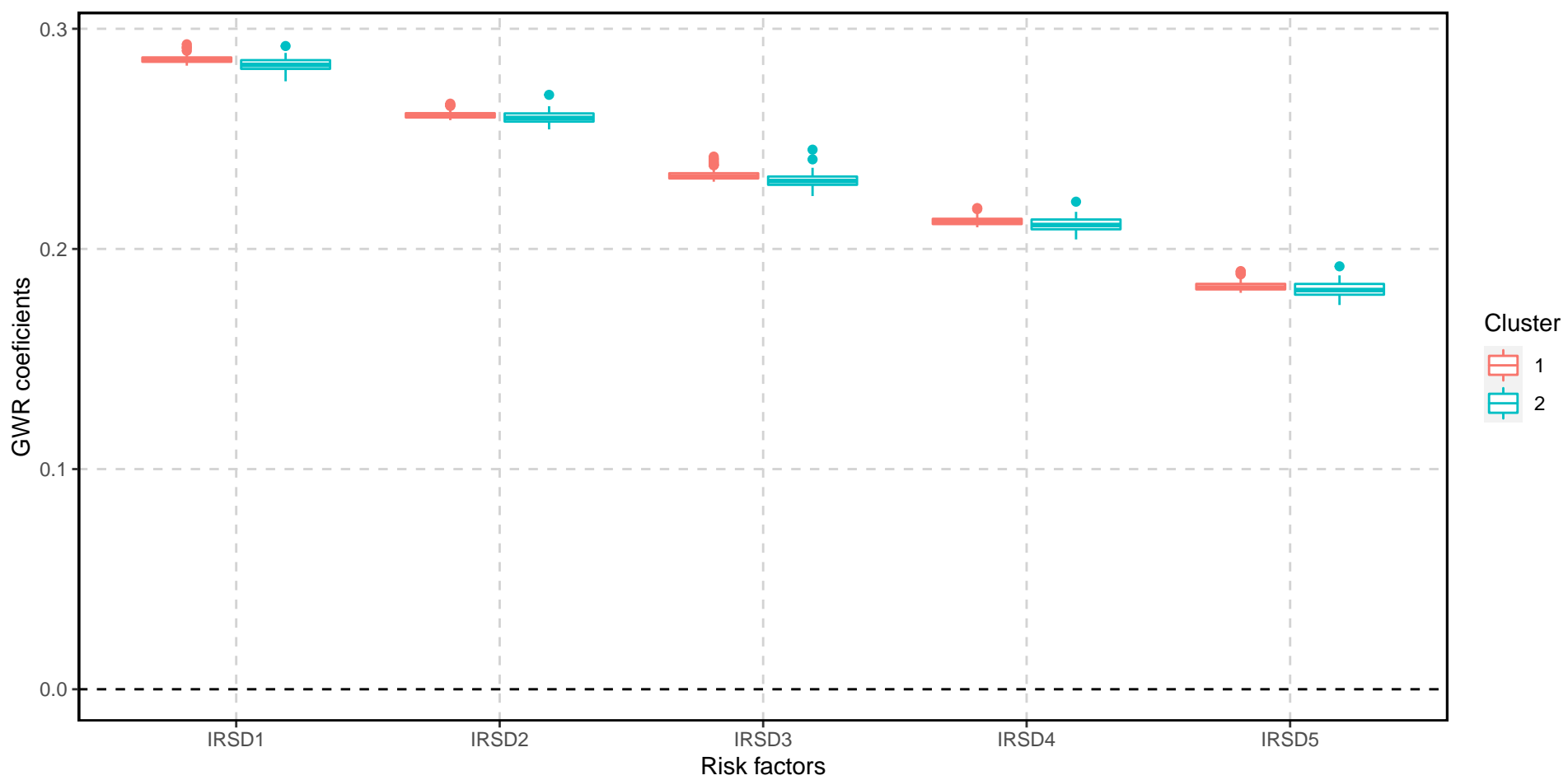

Supplement: S1 Data — (ZIP) [file pone.0285409.s007.zip › Appendix/S2_fig_3.pdf]

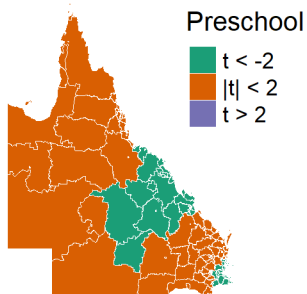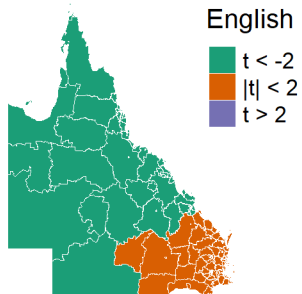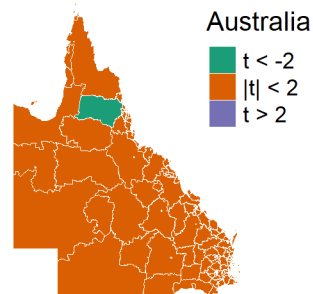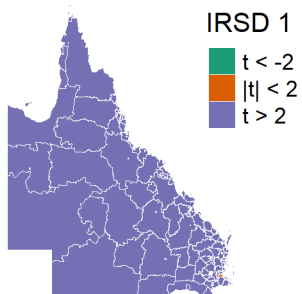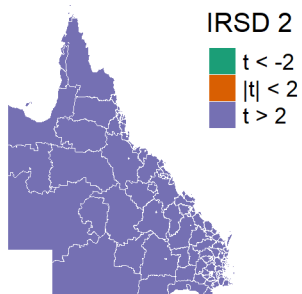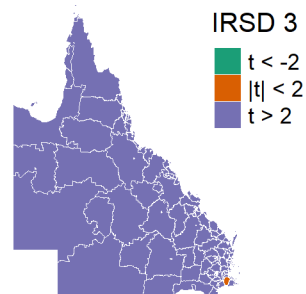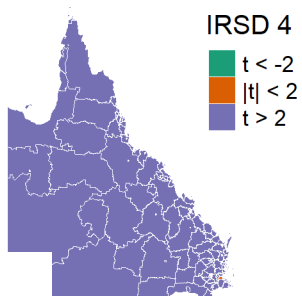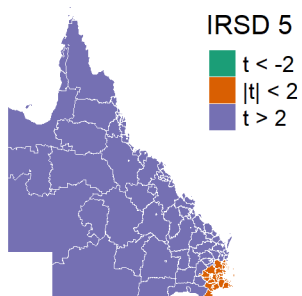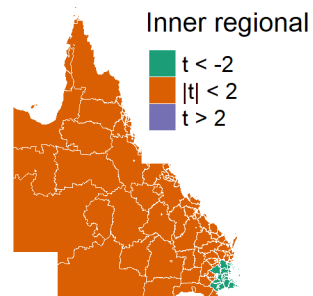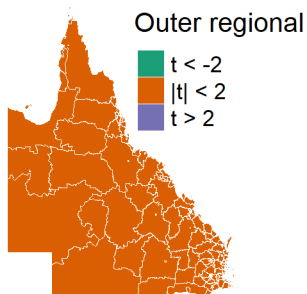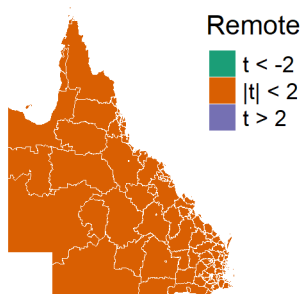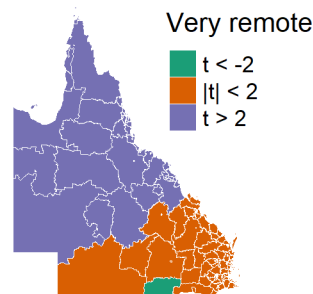

Supplement: S1 Data — (ZIP) [file pone.0285409.s007.zip › Appendix/S2_fig_4.pdf]

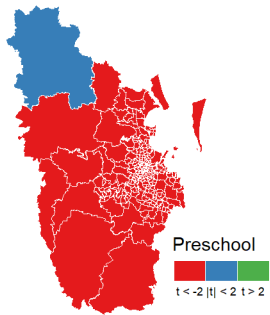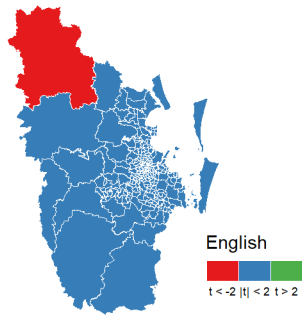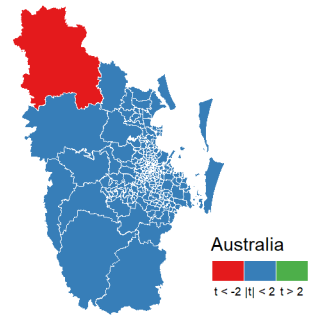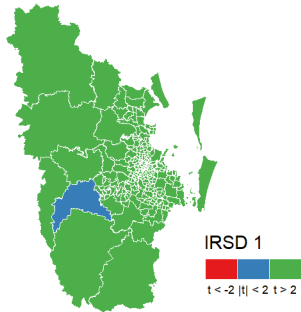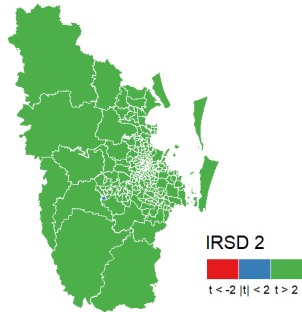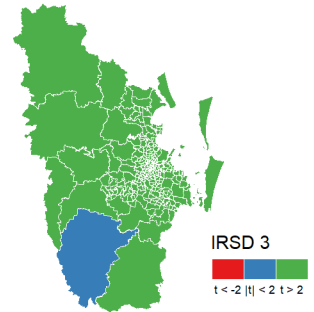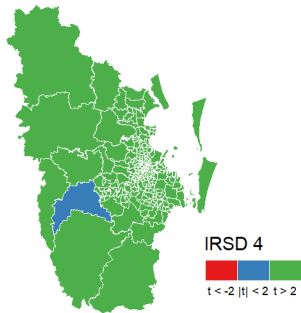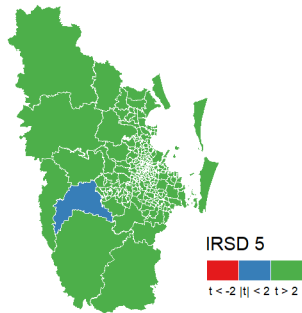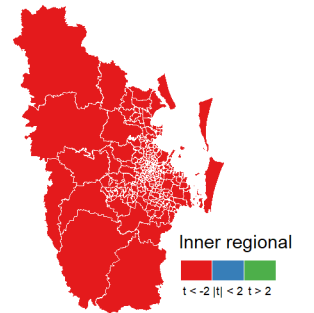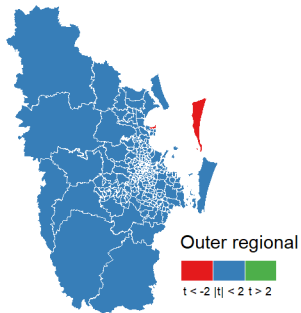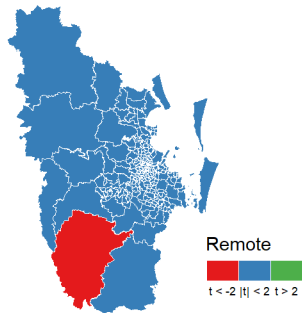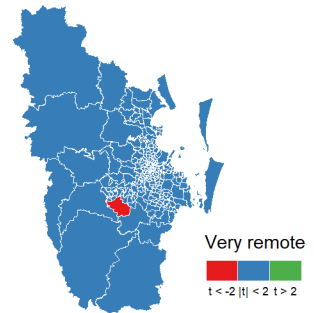

Supplement: S1 Data — (ZIP) [file pone.0285409.s007.zip › Appendix/S2_fig_5.pdf]

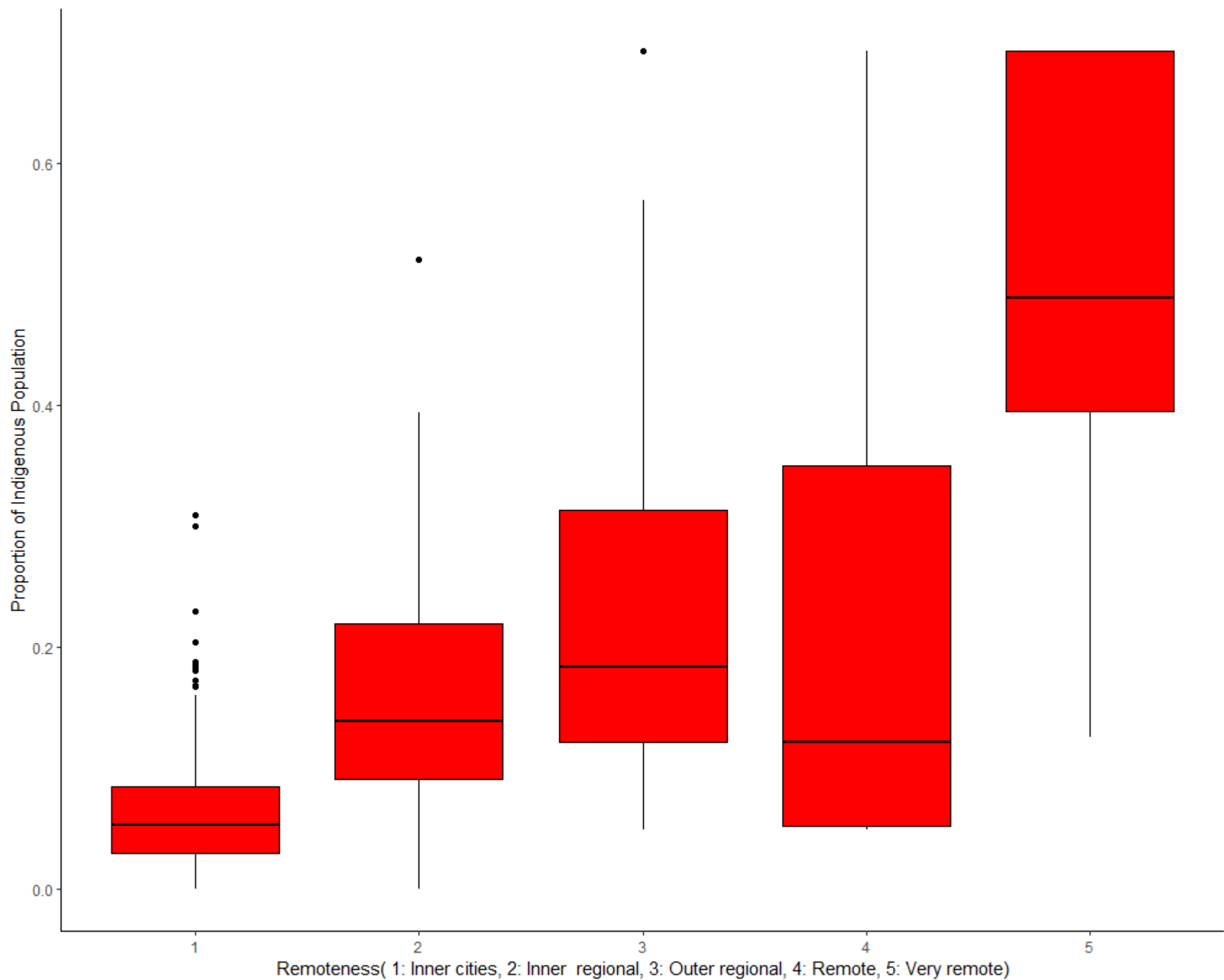

Supplement: S1 Data — (ZIP) [file pone.0285409.s007.zip › Appendix/S4_fig_1.pdf]

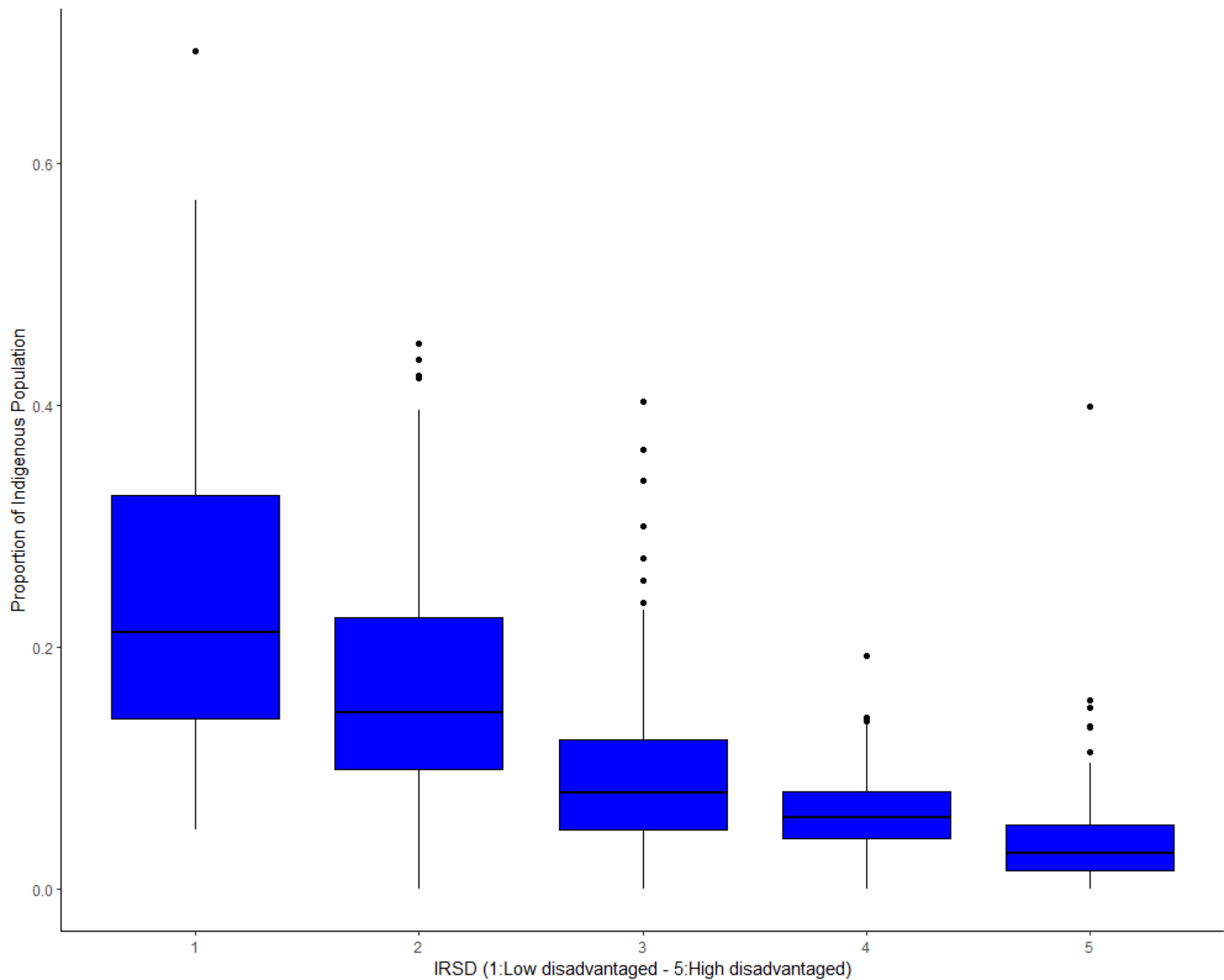

Supplement: S1 Data — (ZIP) [file pone.0285409.s007.zip › Appendix/S4_fig_2.pdf]

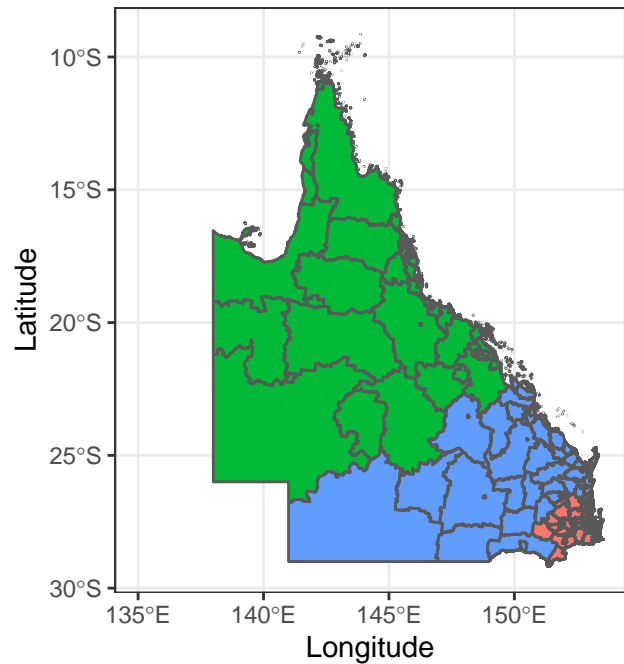

K-means clustering 1 2 3

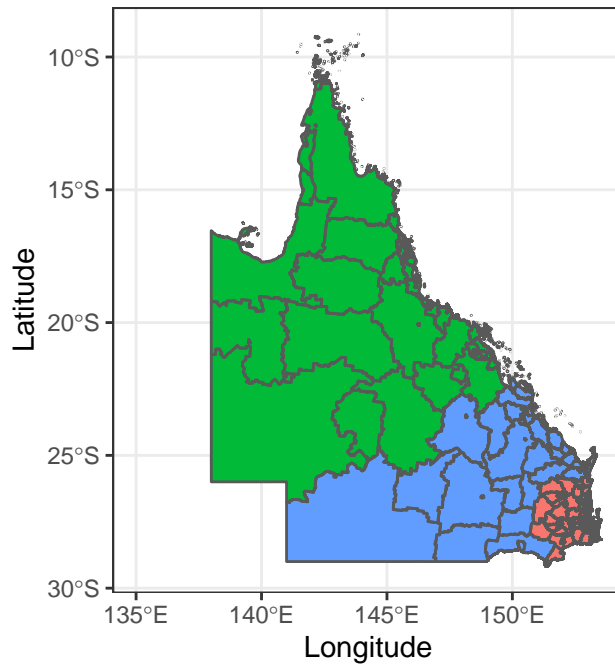

hirechical clustering 1 2 3

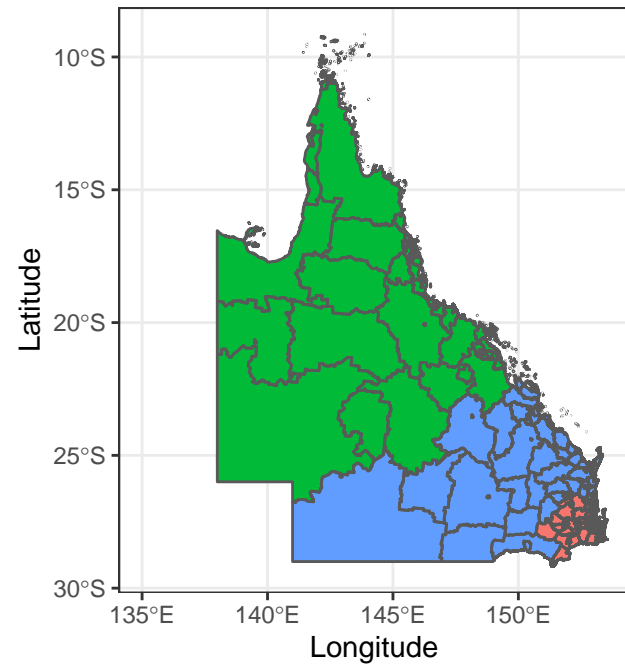

PAM clustering 1 2 3

Supplement: S1 Data — (ZIP) [file pone.0285409.s007.zip › Appendix/S5_fig_1.pdf]
